# Ionomic and proteomic changes highlight the effect of silicon supply on the nodules functioning of *Trifolium incarnatum* L.

**DOI:** 10.3389/fpls.2024.1462149

**Published:** 2024-11-06

**Authors:** Raphaël Coquerel, Mustapha Arkoun, Jacques Trouverie, Benoit Bernay, Philippe Laîné, Philippe Etienne

**Affiliations:** ^1^ UFR des Sciences, Université de Caen Normandie, INRAE, UMR 950 EVA, Caen, France; ^2^ Laboratoire de Nutrition Végétale, Centre Mondial d’Innovation-Groupe Roullier, Saint-Malo, France; ^3^ Université de Caen Normandie, Plateforme Proteogen, US EMerode 4206, Caen, France

**Keywords:** ionomic analysis, nodules, proteomic analysis, silicon, *Trifolium incarnatum* L

## Abstract

**Introduction:**

Numerous studies have reported the beneficial effects of silicon (Si) in alleviating biotic or abiotic stresses in many plant species. However, the role of Si in Fabaceae facing environmental stress is poorly documented. The aim of this study is to investigate the effect of Si on physiological traits and nodulation efficiency in *Trifolium incarnatum* L.

**Methods:**

Si was supplied (1.7 mM in the form of Na_2_SiO_3_) plants inoculated with *Rhizobium leguminosarum* bv *trifolii* and plant physiological traits and nodule ionomic and molecular traits were monitored over 25 days.

**Results:**

Si supply promoted shoot biomass, the quantity of both Si and N in roots and shoots, and the number, biomass and density of nodules and their nitrogenase abundance which contribute to better dinitrogen (N_2_) fixation. Ionomic analysis of nodules revealed that Si supply increased the amount of several macroelements (potassium, phosphorus and sulfur) and microelements (copper, zinc and molybdenum) known to improve nodulation efficiency and N_2_ fixation. Finally, comparative proteomic analysis (+Si *versus* -Si) of nodules highlighted that Si modulated the proteome of both symbionts with 989 and 212 differentially accumulated proteins (DAPs) in the infected host root cells and their symbiont bacteria, respectively.

**Discussion:**

Among the DAPs, the roles of those involved in nodulation and N_2_ fixation are discussed. For the first time, this study provides new insights into the effects of Si on both nodular partners and paves the way for a better understanding of the impact of Si on improving nodule function, and more specifically, on the nodules’ N_2_-fixing capacity.

## 1 Introduction

Silicon (Si), the second most abundant element in the Earth’s crust (McLeod and Shaulis, 2018), has recently gained attention due to its beneficial effects on plant growth and stress alleviation. Over the last ten years, interest has grown in gaining a deeper understanding of the role of Si in plants from both fundamental and applied points of view, as evidenced by the increasing number of research papers and review articles on the subject (Pavlovic et al., 2021). Although Si is not considered an essential element for plants, all studies have highlighted a beneficial effect of Si that only manifests itself when plants are subjected to biotic (Verma et al., 2021) and abiotic stress (Conceição et al., 2019; Sena et al., 2022). In particular, several studies have demonstrated that Si supply alleviates stress in plants subjected to nutritional deficiencies, such as N, P, K and S (Haddad et al., 2018; Pavlovic et al., 2021; Laîné et al., 2022, Kandhol et al., 2024). These positive effects related to Si supply include mechanical (e.g. cell wall strengthening) and physiological changes (e.g. delayed leaf senescence and/or regulation of root transporters) (Markovich et al., 2017; Etienne et al., 2021; Laîné et al., 2022), as well as transcriptomic changes in leaves and roots (Haddad et al., 2019; Etienne et al., 2021) and/or proteomic modifications (e.g. increases in enzymes involved in the detoxification of reactive oxygen species) (Chung et al., 2020; Akhter et al., 2022; Kandhol et al., 2024).

Nitrogen is an essential macronutrient for plant growth and development, and is usually considered a key factor for ensuring the yield and seed quality of many crops (Rütting et al., 2018). While the N nutrition of most plant species relies entirely on their ability to take up inorganic N from the soil solution, Fabaceae also have the ability to fix atmospheric N_2_ thanks to root nodules resulting from a symbiotic association with soil rhizobia (Lindström and Mousavi, 2020). Several studies have shown that the number of root nodules and their fixation capacity are differentially modulated as a function of the inorganic nitrogen availability in the soil. Indeed, Xia et al., 2017 reported that, in soybean, root nodulation was suppressed by high N (>50 mg L^-1^) whereas it was accelerated by low N concentrations (<50 mg L^-1^). Finally, in a recent study, Ke et al. (2024) reported that a total N-deprivation had no effect on root nodulation or nitrogenase activity in soybean. In addition, N_2_ fixation is also impaired by various abiotic stress conditions such as other macronutrient deficiencies like potassium (Liu et al., 2022), phosphorus (Sulieman and Tran, 2015; Yao et al., 2024) and sulfur (Varin et al., 2010; Coquerel et al., 2023), or drought stress (Freitas et al., 2022) and saline stress (Nitawaki et al., 2021), which lead to a decrease in the number of nodules and nitrogenase content.

Recent studies indicate positive effects of Si supply on the nodulation efficiency of plants subjected to stress (El Moukhtari et al., 2022) or non-stress conditions (Putra et al., 2021, 2022). For example, several studies report that Si supply improves the number of nodules and the level of N_2_ fixation in M*edicago sativa* L. and *Trigonella foenum-graecum* L. subjected to salt stress (El Moukhtari et al., 2021; Lamsaadi et al., 2023) and in *Trifolium incarnatum* L. grown under sulfur deficiency (Coquerel et al., 2023). However, to date, no study has investigated the effect of Si supply on nodulation efficiency and N_2_ fixation capacity in plants depending on N_2_ atmospheric as sole N source.

This study aimed to investigate the effects of Si on physiological traits such as shoot and root biomasses, total N and Si content, N_2_ fixation capacity, nodulation parameters (nodule biomass, number, density and their nitrogenase content) in *Trifolium incarnatum* L inoculated with *Rhizobium leguminosarum* bv *trifolii* for a period of 25 days. In addition, an ionomic approach and a fine comparative proteomic analysis (+Si *versus* -Si) that distinguished the two nodule symbionts were carried out to decipher the beneficial effects of Si on nodulation efficiency and N_2_-fixing capacity in particular.

## 2 Materials and methods

### 2.1 Plant growth conditions and experimental design

**Figure 1:**
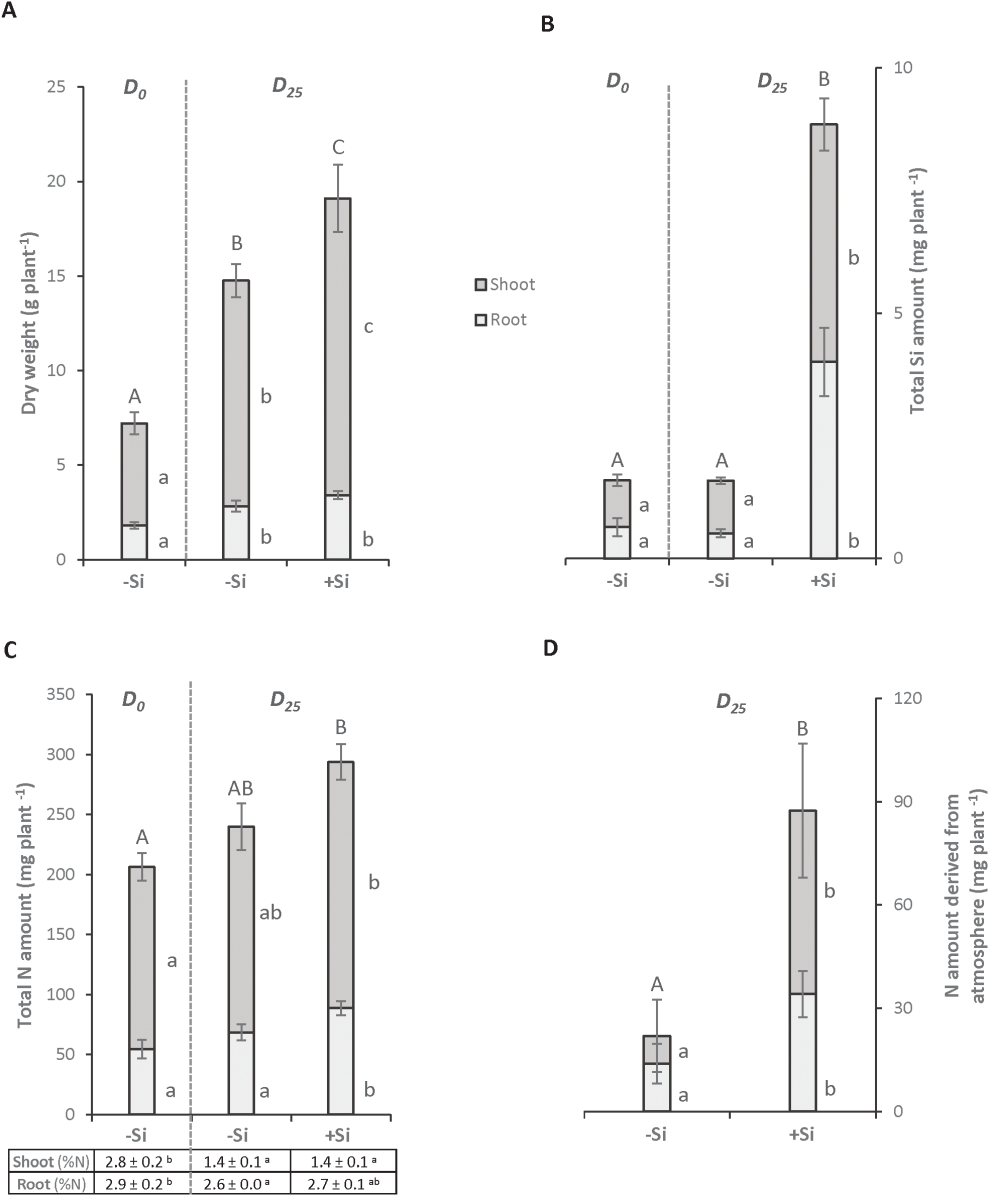
Total dry weight **(A)**, total Si amount **(B)** and total N amounts and concentrations **(C)** in *Trifolium incarnatum* L. plants grown with (+Si; 1.7 mM) or without (-Si; 0 mM) silicon for 25 days (D0 to D25). Table **(C)** indicates N concentration (in % of DW) in each plant compartment. The total N amount derived from the atmosphere **(D)** is calculated by subtracting the total amount of N in the plants at D25 and D0. Different letters indicate that the mean (± S.E.) obtained for each parameter for the whole plant (uppercase letters) or for each compartment (lowercase letters) is significantly different (p < 0.05).

The experimental design is summarized in **Figure 1**. Seeds of *Trifolium incarnatum* L. were germinated on perlite over deionized water (Génard et al., 2017a) for four days in the dark in a greenhouse. When the first leaves emerged, seedlings were transferred to natural light conditions and supplied for one and a half weeks with nutrient solution containing: KNO_3_ (1 mM), KH_2_PO_4_ (0.25 mM), KCl (1 mM), CaCl_2_ (3 mM), MgSO_4_ (0.5 mM), EDTA-2NaFe (0.2 mM), H_3_BO_3_ (14 µM), MnSO_4_ (5 µM), ZnSO_4_ (3 µM), CuSO_4_ (0.7 µM), (NH_4_)_6_Mo_7_O_24_ (0.7 µM), CoCl_2_ (0.1 µM). Then, the seedlings were transplanted into hydroponics tanks (20 L, nine plants per tank) containing the nutrient solution described above and inoculated with *Rhizobium leguminosarum* bv *trifolii* (strain T354, MSDJ1056, Mazurier, 1989; Génard et al., 2017b) previously grown in YEM liquid medium (Gulati, 1979) for 72 h. After two weeks (D0), when plants were sufficiently developed and nodulated, the KNO_3_ was removed from nutrient solution allowing plants to use atmospheric N only. Then, plants were separated into two sets fed with the same nutrient solution as previously described and supplemented (+Si) or not (-Si) with 1.7 mM of Si in the form of Na_2_SiO_3_ for 25 days. To compensate the sodium provided by the Si supply, 3.4 mM NaCl was added to the nutrient solution of -Si plant set. All nutrient solutions, continuously aerated with a compressed air bubbling system, were replaced every three days. At each solution renewal, 10 mL of liquid *Rhizobium leguminosarum* bv *trifolii* culture reaching OD of 0.9 (Λ=600 nm) was added to each tank. Every day, the pH of the nutrient solution was monitored and adjusted if necessary to 5.8 ± 0.2. Throughout the culture duration, light was supplied by high pressure sodium lamps (Philips, MASTER GreenPower T400W) with photosynthetically active radiation of 450 μmol photons·m^−2·^s^−1^ at canopy height.

At D0 and D25, plants were harvested, the nodules were counted, manually separated from roots, weighed and frozen at -80°C. In addition, shoots and roots were separated and weighed. Subsequently, an aliquot of each plant compartment was placed in liquid nitrogen and stored at -80°C and the rest was dried at 60°C for 72h. After dry weight (DW) determination, samples were ground to perform the elemental analyses.

### 2.2 Determination of macroelement and microelement concentrations and N amounts derived from the atmosphere

To determine the total N concentration, 1.5 mg of each dried powder was precisely weighed and placed into tin capsules before analysis with a continuous flow isotope ratio mass spectrometer (IRMS, Horizon, NU Instruments, Wrexham, UK) linked to a C/N/S analyzer (EA3000, Euro Vector, Milan, Italy). The total N amount (Ntot) in each organ or in the whole plant was calculated as:


$$Ntot=\;\frac{\%N\;x\;DW\;\left(or\;FW\right)}{100}$$

In addition, the N amount derived from the atmosphere (*Natm*) during the period was calculated as: *Natm* = *Ntot at D*25 − *Ntot at D*0

The N concentration in nodules (n=5) has been estimated by dividing the protein concentration in the nodules (see below) by the conversion factor 6.25 (Marks et al., 1985).

For total Si concentration determination, approximately 1 g of the ground dry root or shoot sample was analyzed with an X-ray-fluorescence spectrometer (XEPOS, Ametek, Berwyn, PA, USA) using calibration curves obtained from international standards. The other macroelement (magnesium: Mg, phosphorus: P, sulfur: S, potassium: K and calcium Ca) and microelement (copper: Cu; molybdenum: Mo, iron: Fe, zinc: Zn; boron: B and cobalt: Co) concentrations were quantified in nodules from 40 mg of fresh powder previously subjected to mineralization before analysis using high-resolution inductively coupled plasma mass spectrometry (HR-ICP-MS, Element 2™, Thermo Fisher Scientific, Bremen, Germany) as previously described by Maillard et al. (2016). Briefly, fresh nodules previously powdered using a mortar containing liquid nitrogen were subjected to microwave acid sample digestion (Multiwave ECO, Anton Paar, les Ulis, France) with 1 mL of concentrated nitric acid (HNO_3_), 250 μL of H_2_O_2_, 900 μL of ultrapure water and 10 μL of internal standard of gallium (10 μg L^–^1) and rhodium (2 μg L^–1^). Digested samples were diluted with ultrapure water to obtain a 2.0% (v/v) solution of HNO_3_ before filtration through a 0.45 μm Teflon filter. Quantification of each element was performed from calibration curves after being corrected from the recovery rate by subtracting the blank and using internal standards (Ga and Rh). The quality of mineralization and quantification was evaluated using certified reference plant material (Citrus leaves, CRM NCS ZC73018, Skylab, Metz, France). For each element, the amount in nodules was calculated by multiplying the concentration by the fresh nodule biomass.

### 2.3 Proteins analysis

#### 2.3.1 Extraction and quantification of proteins from nodules

Total proteins were extracted from nodules using the protocol previously described by Varin et al. (2010). Briefly, freshly isolated nodules were ground in liquid nitrogen in a mortar. Approximately 30 mg of powder was mixed in 2 mL of cold acetone containing 10% trichloroacetic acid (w/v) and centrifuged at 16,000 g (4 min at 4°C). The supernatant was then removed and the pellet was resuspended with 1.75 mL of ammonium acetate (0.1 M)/methanol (80%) buffer to precipitate the proteins. After centrifugation at 16,000 g (3 min at 4°C), the supernatant was removed and the pellet was washed with 1 mL of acetone (80%) and centrifuged again. The resulting pellet was resuspended with 800 µL of phenol (pH 8) and 800 µL of sodium dodecyl sulfate (SDS) buffer [30% saccharose (w/v), 2% SDS (w/v), 0.1 M TRIS-HCl (w/v), 0.5% β-mercaptoethanol (v/v), pH 8] then centrifuged at 16,000 g (10 min at 4°C). The upper phenolic phase was recovered and 800 µL of ammonium acetate (0.1 M)/methanol (80%) buffer was added. After storing overnight at -20°C, the mixture was centrifuged at 16,000 g (10 min at 4°C). Then, the pellet was washed with 1 mL of methanol (100%) and after centrifugation at 16,000 g (10 min at 4°C) it was washed a second time with 1 mL of acetone (80%). The pellet was resuspended with a buffer containing dithiothreitol (DTT, 11.11 mM), thiourea (2.22 M), urea (6.66 M) and Tris buffer 2 M (33.33 mM) and used for the determination of the protein concentration using the Bradford method (Bradford, 1976).

#### 2.3.2 Quantification of nitrogenase abundance in nodules

The abundance of nitrogenase in nodules was determined after Western blotting of 20 µg of total proteins extracted from isolated nodules previously separated by SDS-PAGE (12% polyacrylamide gels at a constant current: 250 V, 75 mA, 1 h). Western blots were carried out as recently described by Coquerel et al. (2023). Briefly, after Western blotting, PVDF membrane was incubated overnight with chicken polyclonal anti-nitrogenase (NifH) from IgG antibodies (Agrisera, SE 911, Vanadas, Sweden; dilution 1:2000). Subsequently, the PVDF membrane was washed four times with TBST (Tris-base 10 mM, NaCl 150 mM, pH 8, 0.1% Tween 20) before incubation with secondary antibody (rabbit anti chicken immunoglobin Y coupled to alkaline phosphatase; dilution 1:6000) for 1 h. The nitrogenase–antibody complex was revealed using a Bio-Rad alkaline phosphatase kit as a single signal at a molecular weight of between 25 and 37 kDa. Western blots were scanned and analyzed using a Millipore Bioimage computerized image analysis system to determine the intensity of signal (U.A.) that was proportional to the nitrogenase abundance in each nodule sample. The nodule nitrogenase concentration (U.A. g^−1^ FW) was calculated by dividing the intensity value by the fresh nodule biomass (20 µg). Then the total abundance of nitrogenase in all the nodules of one plant was estimated by multiplying the nitrogenase concentration by total nodule biomass. The nitrogenase abundance at D25 was expressed relative to the nitrogenase abundance determined at D0 (100%).

#### 2.3.3 Proteomic mass spectrometry sample preparation and protein identification

Five µg of each nodule protein extract were prepared using the modified gel-aided sample preparation protocol described by Fischer and Kessler (2015). Samples were digested with trypsin/Lys-C overnight at 37°C. For nano-LC fragmentation, peptide samples were first desalted and concentrated onto a µC18 Omix (Agilent, Santa Clara, USA) before analysis. The chromatography step was performed on a NanoElute (Bruker Daltonics, Massachusetts, USA) ultra-high-pressure nano flow chromatography system. Approximatively 50 ng of each peptide sample were concentrated onto a C18 pepmap 100 (5 mm x 300 µm i.d.) precolumn (Thermo Scientific, Dreieich, Germany) and separated at 50°C onto a reversed phase Reprosil column (25 cm x 75 μm i.d.) packed with 1.6 μm C18 coated porous silica beads (Ionopticks). Mobile phases consisted of 0.1% formic acid, 99.9% water (v/v) (A) and 0.1% formic acid in 99.9% ACN (v/v) (B). The nanoflow rate was set at 300 nl/min, and the gradient profile was as follows: from 2 to 15% B within 15 min, followed by a second increase to 25% B within 15 min and a third one to 37% B within 5 min and final to 95% within 7 min and re-equilibration. MS experiments were carried out on a TIMS-TOF pro mass spectrometer (Bruker Daltonics, Massachusetts, USA) with a modified nano electrospray ion source (CaptiveSpray, Bruker Daltonics, Massachusetts, USA). A 1400 spray voltage with a capillary temperature of 180°C was typically employed for ionizing. MS spectra were acquired in the positive mode in the mass range from 100 to 1700 m/z and a 0.75 to 1.30 1/k0 window. In the experiments described here, the mass spectrometer was operated in PASEF DIA mode with exclusion of single charged peptides. The DIA acquisition scheme consisted of 24 variable windows ranging from 300 to 1000 m/z. Database searching and LFQ quantification (using XIC) were performed using DIA-NN (version 1.6.2) (Demichev et al., 2020). Updated UniProt *Rhizobium leguminosarum* and *Trifolium repens* databases were used for library-free search generation. For RT prediction and extraction mass accuracy, we used the default parameter 0.0, which means DIA-NN performed automatic mass and RT correction. The top six fragments (ranked by their library intensities) were used for peptide identification and quantification. The variable modifications allowed were as follows: Nterminal-acetylation and oxidation (M). In addition, C-propionoamide was set as a fixed modification. “Trypsin/P” was selected. Data were filtered according to a False Discovery Rate (FDR) of 1%. Cross-run normalization was performed using RT-dependent.

#### 2.3.4 Identification and GO enrichment analysis of differentially accumulated proteins

To quantify the abundance of differentially accumulated proteins (DAPs) under Si supply, data from DIA-NN were then analyzed using the DEP package from R. Briefly, proteins that were identified in two out of three replicates of at least one condition were filtered, missing data were input from Perseus software using a normal distribution, and differential enrichment analysis was based on linear models and empirical Bayes statistics. The dataset was subjected to analysis of variance (Anova) using Perseus. To determine DAPs, a 1.5-fold variation in the relative abundance (Fold-Change; FC) and a 0.05 FDR from multiple-sample tests or 0.01 p-value from two-sample tests were used. All these proteomics data have been deposited to the ProteomeXchange Consortium (Perez-Riverol et al., 2022) *via* the PRIDE partner repository with the data-set identifier IPX0008420001. Gene Ontology (GO) enrichment analysis for *Trifolium* DAPs was initially performed using ShinyGO (version V.08; Ge et al., 2020) based on the Ensemble Plants gene ID (Ensembl Release 104, archived on Jan. 5, 2024) and then ReviGO (version 1.8.1; Supek et al., 2011) based on the Gene Ontology database (go.obo; January 17, 2024) and the UniProt-to-GO mapping database from the EBI GOA project (goa_uniprot_gcrp.gaf.gz; February 9, 2024) to summarize enriched GO by removing redundant terms (medium allowed similarity). Since no GO enrichment analysis software could be found for *Rhizobium leguminosarum*, ClueGO (version 2.5.10; Jun 21, 2023; Bindea et al., 2009), which is based on *Rhizobium leguminosarum* GO annotation (release of July 7, 2019), was used to create functionally grouped GO term networks using Kappa statistics (Kappa Score=0.4).

### 2.4 Statistical analysis

All data are indicated as the mean ± S.E (n=10 for plant samples, n=5 for nodule samples and n=3 for proteomic analysis). Statistical analyses were performed using R software (version 4.2.0: R Core Team, 2022). Significant differences between harvest dates and treatments were determined using Student’s t-test.

## 3 Results

### 3.1 Effect of Si supply on growth, total N and Si amounts in *Trifolium incarnatum* L.

Regardless of the Si treatment (-Si or +Si), after 25 days both plants showed significantly higher root and shoot biomasses than plants harvested at D0. In addition, the total biomass of +Si plants was significantly higher than this of -Si plants. This increase can be explained by a rise in shoot biomass (15.70 ± 1.78 g and 11.93 ± 0.87 g for +Si and -Si plants, respectively) (**Figure 1A**). As expected, the addition of Si to the nutrient solution led to a significant 5- and 8-fold increase in the Si amounts in shoots and roots, respectively. This resulted in a Si accumulation in +Si whole plants of about 4.5-fold relative to control plants (-Si; **Figure 1B**). A Si supply between D0 and D25 led to a significant increase in the N amount (from 206 ± 19 mg to 293 ± 12 mg) in +Si plants, while no significant difference was observed in -Si plants (**Figure 1C**). Since the N concentration in each compartment is similar between -Si and +Si plants (**Figure 1C**) at D25, this increase in N amount between D0 and D25 is explained by the increase in shoot biomass of +Si plants (**Figure 1A**). Moreover, the increase in the amount of N in +Si plants is due to an enhanced capacity to fix atmospheric dinitrogen (N_2_), which was highlighted by the difference in the N amounts in whole plants between D0 and D25 (**Figure 1D**).

### 3.2 Effect of Si supply on nodule parameters

**Figure 2:**
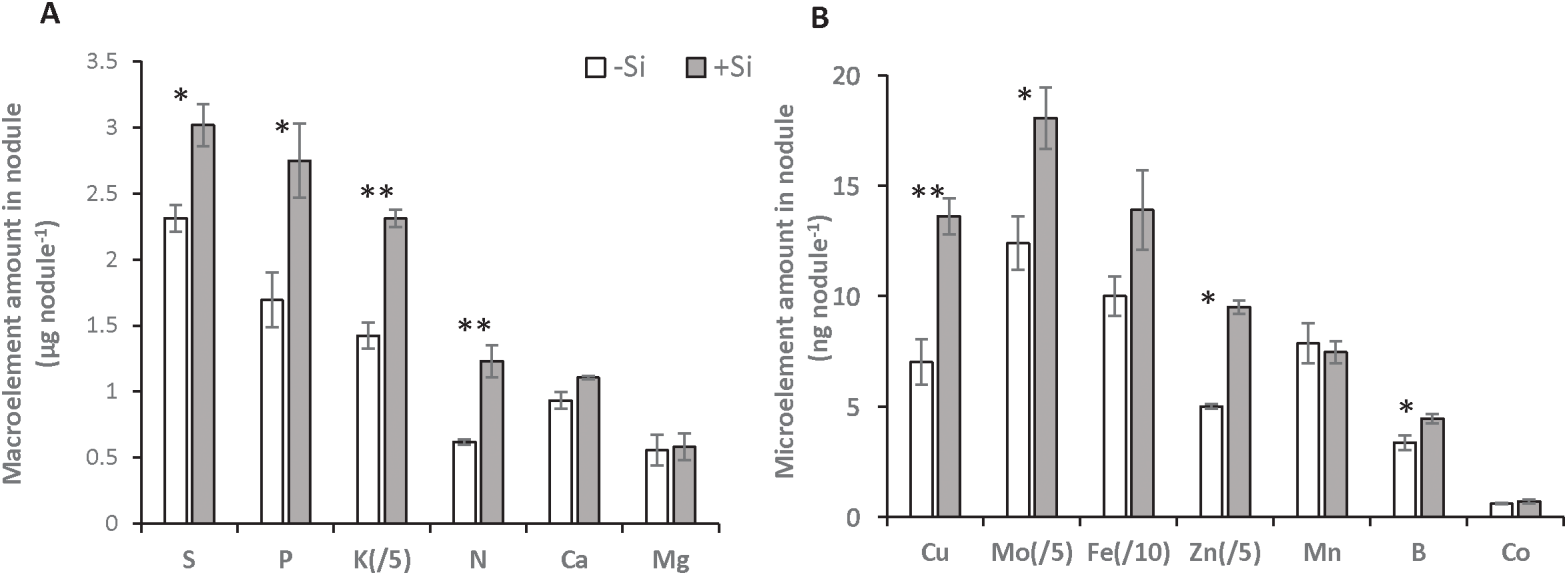
Macroelement **(A)** and microelement **(B)** amounts in nodules from *Trifolium incarnatum* L. plants grown with (+Si; 1.7 mM) or without (-Si; 0 mM) silicon for 25 days. The amount of Fe is divided by 10 and the amounts of K, Mo and Zn are divided by 5. Asterisks indicate that means ± S.E. (n=4) obtained for one element are significantly different between both Si treatments (*p < 0.05 and **p < 0.01).

Compared with D0, -Si plants did not affect nodule parameters (total number per plant, density and biomass) or their relative nitrogenase abundance (**Table 1**). Compared with -Si plants, by D25 the supply of Si (+Si) had increased the total number of nodules per plant (by about 2.3-fold, from 1469 ± 252 to 3402 ± 339), nodule root density (by about 1.5-fold, from 64 ± 8 to 84 ± 5 nodule g^-1^ of FW root) and the nodules’ relative nitrogenase abundance (by about 2.2-fold, from 136 ± 1 to 303 ± 20%) (**Table 1**). Composition analysis of the macroelements (S, P, K, N, Ca and Mg) and microelements (Cu, Mo, Fe, Zn, Mn, B and Co) in nodules revealed that supplying Si was associated with a significant increase in total amount of S, P, K Cu, Mo, Zn and B without changing their concentration (**Figure 2**; **Supplementary Table SD1**). An increase in both the total N amount (from 0.62 ± 0.02 to 1.23 ± 0.12 µg nodule^-1^) and its concentration (from 189.8 ± 8.5 to 324.4 ± 36.5 µg g^-1^ of nodule FW) was observed in nodules of +Si treated plants compared to -Si plants. For the other macroelements (Ca and Mg) and microelements (Fe, Mn and Co), the total amount in nodules remained similar, regardless the Si treatment (**Figure 2**; **Supplementary Table SD1**).

**Table 1:** Changes in the number, biomass, density and relative abundance of nitrogenase in nodules of *Trifolium incarnatum* L. supplied (+Si) or not supplied (-Si) with Si (D0 to D25). Values are means ± SE *(n=5*).

		Number of nodules (nodules plant^-1^)	Biomass of a single nodule (mg FW)	Nodule density (nodule g^-1^ of FW root)	Relative abundance of nitrogenase in nodule (%)
D_0_	-Si	*1461.97* *(± 273) a*	*1.59* *(± 0.11) a*	*66.15* *(± 14.61) a*	*100* *(± 34.22) a*
D_25_	-Si	*1469.07* *(± 252) a*	*1.75* *(± 0.14) a*	*64.05* *(± 8.25) a*	*136.08* *(± 1.53) a*
	+Si	*3402.76* *(± 339) b*	*2.61* *(± 0.24) b*	*84.57* *(± 4.87) b*	*303.37* *(± 20.62) b*

Different letters indicate significant differences between harvest dates (D0 and D25) and treatments (p < 0.05).

### 3.3 Effect of Si supply on the whole-nodule proteome

**Figure 3:**
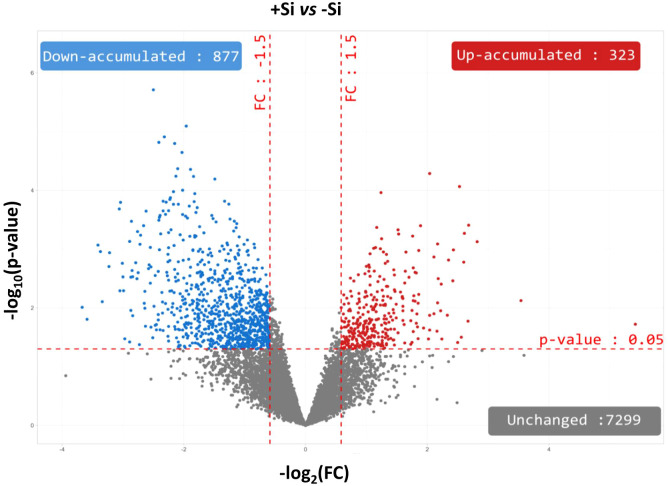
Volcano plots representing all proteins identified in nodules of *Trifolium incarnatum* L. supplied (+Si) or not (-Si) with Si for 25 days. The *X*-axis indicates the log_2_FoldChange (FC) and the *Y*-axis specifies the -log_10_(p-value) of all nodule proteins identified. All proteins with a FC > 1.5 or < -1.5 and a *p-*value < 0.05 are considered as differentially accumulated proteins (DAPs) in response to Si supply. Proteins with unchanged accumulation are shown as grey dots and up- and down-accumulated proteins are indicated by red and blue dots, respectively.

**Figure 4:**
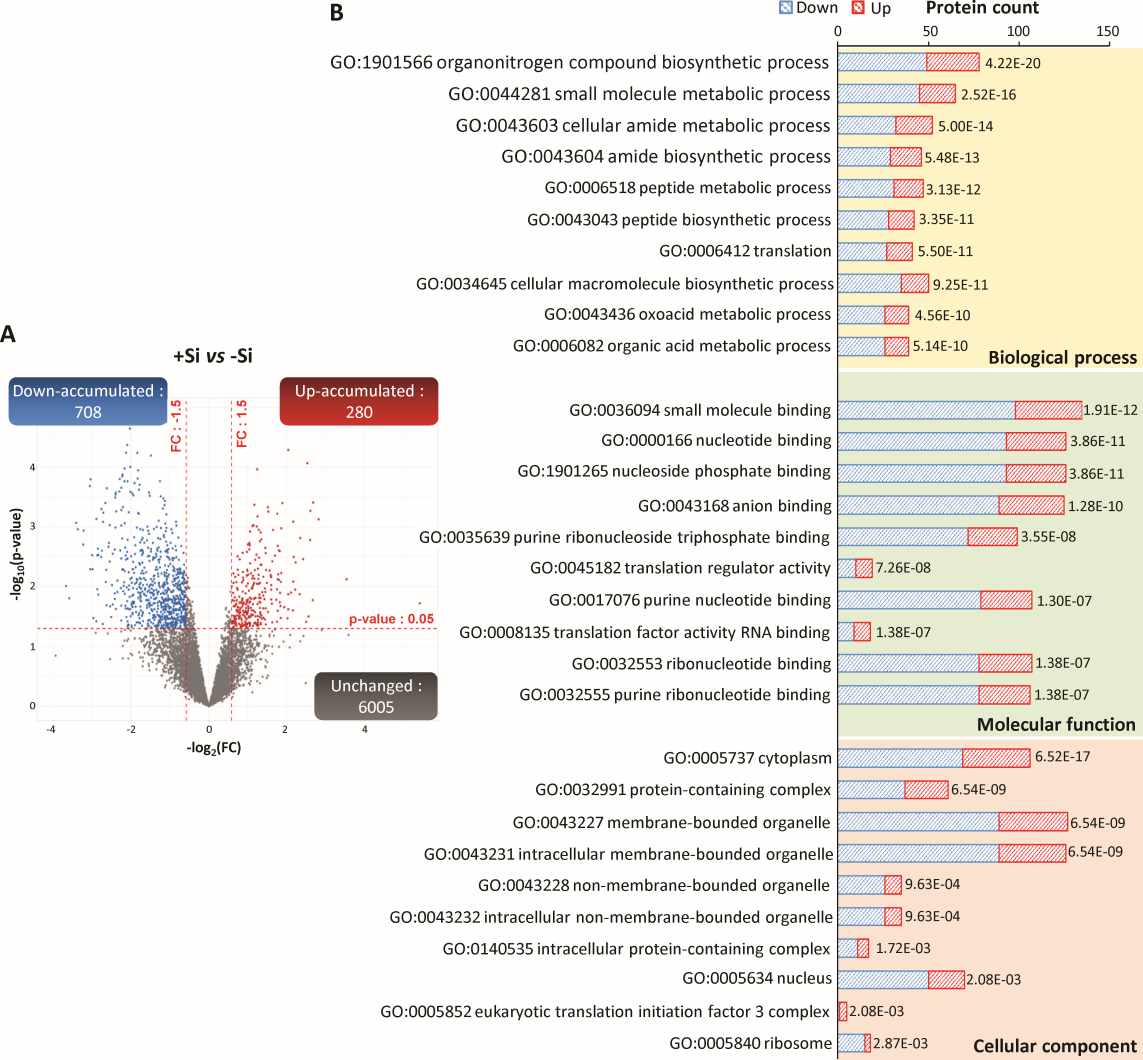
Proteins of infected root cells identified in nodules of *Trifolium incarnatum* L. supplied (+Si) or not (-Si) with Si for 25 days. **(A)** Volcano plots representing all proteins belonging to infected root cells identified in nodules. The *X*-axis indicates the log2FoldChange (FC) and the *Y*-axis specifies the -log_10_(p-value) of identified proteins. All proteins with a FC > 1.5 or < -1.5 and a p-value < 0.05 are considered as differentially accumulated proteins (DAPs) in response to Si supply (+Si *versus* control -Si). Proteins with unchanged accumulation are shown as grey dots and up- and down-accumulated DAPs are indicated by red and blue dots, respectively. **(B)** Top ten enriched GO terms from the GO enrichment analysis of nodule DAPs belonging to infected root cells. Yellow, green and red areas indicate biological process (BP), molecular function (MF) and cellular component (CC) categories, respectively. The y-axis indicates the number of up- (red) or down-accumulated (blue) DAPs in each GO term. The numbers beside each histogram correspond to an FDR calculated based on a nominal *p-*value. For each GO category (BP, MF or CC), enriched GO terms are ranked according to increasing FDR values.

**Figure 5:**
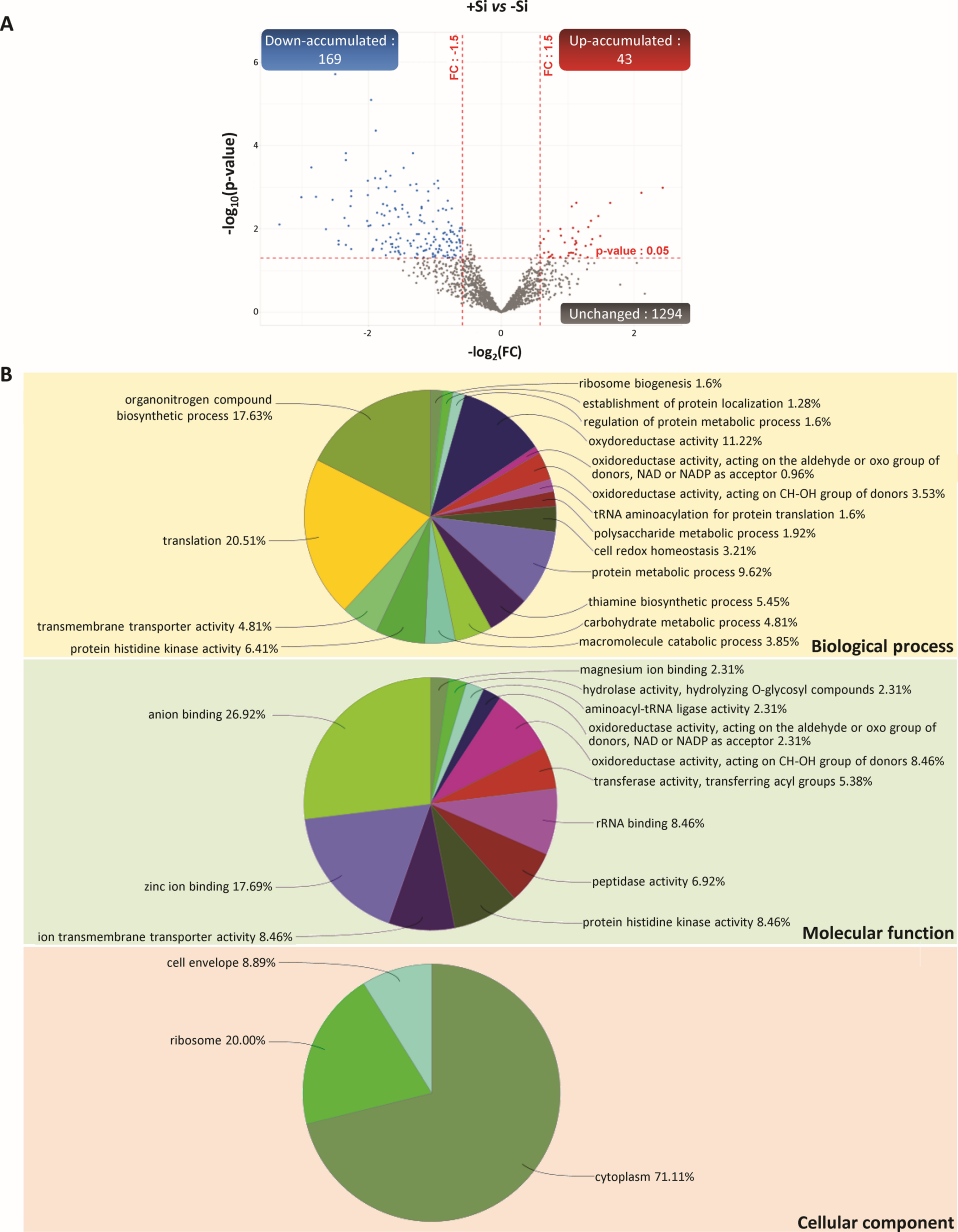
Proteins of bacteria (*Rhizobium leguminosarum* bv *trifolii*) identified in nodules of *Trifolium incarnatum* L. supplied (+Si) or not (-Si) with Si for 25 days. **(A)** Volcano plots representing all proteins identified in nodules and belonging to bacteria. The *X*-axis indicates the log_2_FoldChange (FC) and the *Y*-axis specifies the -log_10_(p-value) of identified proteins. All proteins with a FC > 1.5 or < -1.5 and a p-value < 0.05 are considered as differentially accumulated proteins (DAPs) in response to Si supply (+Si *versus* control -Si). Proteins with unchanged accumulation are shown as grey dots and up- and down-accumulated proteins are indicated by red and blue dots, respectively. **(B)** Bacterial DAPs were grouped into GO term networks according to their kappa scores.

In order to better understand the impact of Si supply on the nodule proteome at D25, proteins were identified in nodules of -Si and +Si plants. Among the 8499 proteins identified, 7299 (i.e. 85,8%) accumulated to similar levels irrespective of the Si treatment, and 1200 were significantly modulated in +Si plants compared to control (-Si) plants (**Figure 3**; **Supplementary SD1**). Thus, 14.2% of the identified proteins corresponded to proteins that were differentially accumulated (DAPs) between +Si and -Si. Among these DAPs, 323 and 877 were up- and down-accumulated by the Si supply, respectively (**Figure 3**; **Supplementary SD1**). In order to refine this global proteomic analysis, the nodule proteins belonging to the infected root cells of *Trifolium incarnatum* L. were distinguished from those belonging to the bacteria (*Rhizobium leguminosarum* bv *trifolii)*. Thus, of the 8499 proteins identified in the nodules, 6993 (i.e. 82.3%) were tagged as proteins of infected root cells (**Figure 4A**; **Supplementary SD1**) and 1506 (i.e. 17.7%) were labelled as proteins of the bacteria (**Figure 5A**; **Supplementary SD1**).

### 3.4 Effect of Si supply on the proteome of infected root cells

Of the 6993 nodular proteins belonging to infected root cells, 6005 had a similar abundance regardless of the Si treatment and 988 were differentially accumulated in response to Si supply (+Si *vs* -Si). Of these DAPs, 708 (i.e. 78% of DAPs) were down-accumulated and 280 (i.e. 22% of DAPs) were up-accumulated in response to Si supply (**Figure 4A**; **Supplementary SD1**). The GO enrichment analysis of these 988 detected DAPs revealed that 711 GO terms were significantly enriched, including 373 related to biological process (BP), 216 to molecular function (MF) and 122 to cellular component (CC) categories (**Supplementary SD1**). The top 10 of each of the GO categories (BP, MF and CC) are shown in **Figure 4B**. For BP, the most enriched GO terms were primarily related to N compound metabolism (GO:19701566, GO:0006518, GO:0043043, GO:0043603, GO:0043604), but also to “small molecule metabolic process” (GO:0044281), “cellular macromolecule biosynthetic process” (GO:0034645), “oxoacid metabolic process” (GO:0043436) and “organic acid metabolic process” (GO:0006082). With regard to MF, the most enriched GO terms were mainly related to nucleotide binding (GO:0032553, GO:0000166, GO:19011265, GO:0017076, GO:0032555, GO:0035639) and translation (GO:0006412, GO:0045182 and GO:000135) but also “small molecule binding” (GO:0036094) and “anion binding” (GO:0043168). Finally, for CC, the main enriched GO terms were related to membrane- and non-membrane-bounded organelle (GO:0043227 and GO:0043228), “intracellular-membrane- and non-membrane-bounded organelle” (GO:0043231 and GO:0043231), “cytoplasm” (GO:005737), “ribosome” (GO:0005840), “nucleus” (GO:0005634), “protein-containing complex” (GO:0032991 and GO:0140535) and “eukaryotic translation initiation factor 3 complex” (GO:0005852).

### 3.5 Effect of Si supply on the bacterial proteome

Among the 1506 nodular proteins belonging to the bacteria (*Rhizobium leguminosarum* bv *trifolii*), 1294 had a similar accumulation irrespective of the Si treatment and 212 were differentially accumulated in response to Si. Among these DAPs, 169 and 43 were down- and up-accumulated in response to Si, respectively (**Figure 5A**; **Supplementary SD1**). Because the usual enrichment analysis tools (such as Shiny GO) do not allow GO enrichment analysis for *Rhizobium leguminosarum* bv *trifolii* proteins, a GO term was assigned to each protein before they were clustered into functional groups for each category (BP, MF and CC) using ClueGO software (**Figure 5B**; **Supplementary SD1**). Regarding BP, most of the DAPs (63.47%) were assigned to functional groups related to “translation” (20.51%), “oxidoreductase process” (18.92% including “oxidoreductase activity” and “cell redox homeostasis”), “organonitrogen compound biosynthetic process” (17.63%) and “protein histidine kinase activity” (6.41%). For the MF category, most of the DAPs (82.67%) were assigned to ion binding (46.92% including “anion binding” (26.92%), “zinc ion binding” (17.69%) and “magnesium ion binding” (2.31%)), “oxidoreductase activity” (10.37%), “tRNA binding” (8.46%), “protein histidine kinase activity” (8.46%) and “ion transmembrane transporter activity” (8.46%). Finally, for the CC category, 71.11%, 20.0% and 8.89% of DAPs were assigned to “cytoplasm”, “ribosome” and “cell envelop”, respectively (**Figure 5B**; **Supplementary SD1**).

## 4 Discussion

The aim of this study was to decipher the effects of silicon supply on the nodulation and N_2_ fixation capacity of *Trifolium incarnatum* L. grown in the presence of N_2_ as the sole N source. In -Si plants, the shoot and root biomasses at D25 were increased compared to D0 plants, while the total amount of N was not significantly different. This result suggests that the amount of N derived from N_2_ fixation during the period (21.5 mg plant^-1^) is sufficient to sustain the growth of plants (**Figure 1**). This finding is consistent with maintenance of the number, biomass, and density of nodules as well as their nitrogenase abundance in –Si plants after 25 days (D25) compared with D0 plants (**Table 1**). This absence of change in nodulation parameters agrees with a recent study reporting that soybean cultivated with a nutrient solution without N for 25 days had numbers of nodules and a nodule biomass similar to plants fed with 2 mM nitrate (Ke et al., 2024). In contrast, +Si plants possessed nodules that were bigger, more numerous and contained higher levels of nitrogenase than -Si plants, leading to an increase in their total N supply due to a sharp rise in their atmospheric N_2_ fixation capacity (from 21.5 to 87.5 mg plant^-1^). All this contributed to improving the growth of +Si plants compared with -Si plants (**Figure 1**, **Table 1**). Taken together, these results highlight the beneficial effect of Si supply on plants, improving nodulation, N_2_ fixation capacity and growth of +Si plants compared to -Si plants. These results are consistent with studies in numerous legume species where supplying Si enhanced the number and size of root nodules as well as the nodules’ N_2_ fixation capacity, and improved growth under biotic (Putra et al., 2022) and other abiotic stresses (El Moukhtari et al., 2022, 2024; Coquerel et al., 2023). In the current study, more refined analyses were carried out to better understand the beneficial effects of Si supply. Ionomic analysis highlighted that supplying Si leads to an increase in several macroelements (such as P and K) and microelements (such as Cu, Zn and B) amounts in nodules (**Figure 2**). Independently, all of these elements are known to have positive effects on nodulation and N_2_ fixation in nodules. As an example, some studies have reported that accumulation of metals such as Cu or Zn but also macroelements like K and P increases the level of N_2_ fixation and the amount of N in plant tissue (Shukla and Yadav, 1982; Premaratne and Oertli, 1994; González-Guerrero et al., 2014; Yao et al., 2022). Moreover, B has been shown to be an important microelement in facilitating establishment of the symbiosis between root cells and bacteria (Bolanos et al., 1996). From the present study it may be suggested that macroelements and microelements, whose quantities increased in nodules, may act individually or synergistically to improve nodulation efficiency (**Table 1**) and/or the N_2_-fixing capacity (**Figure 1D**) of +Si plants, consequently increasing the N amount in nodules (**Figure 2A**) as well as whole +Si plants (**Figure 1C**).

**Figure 6:**
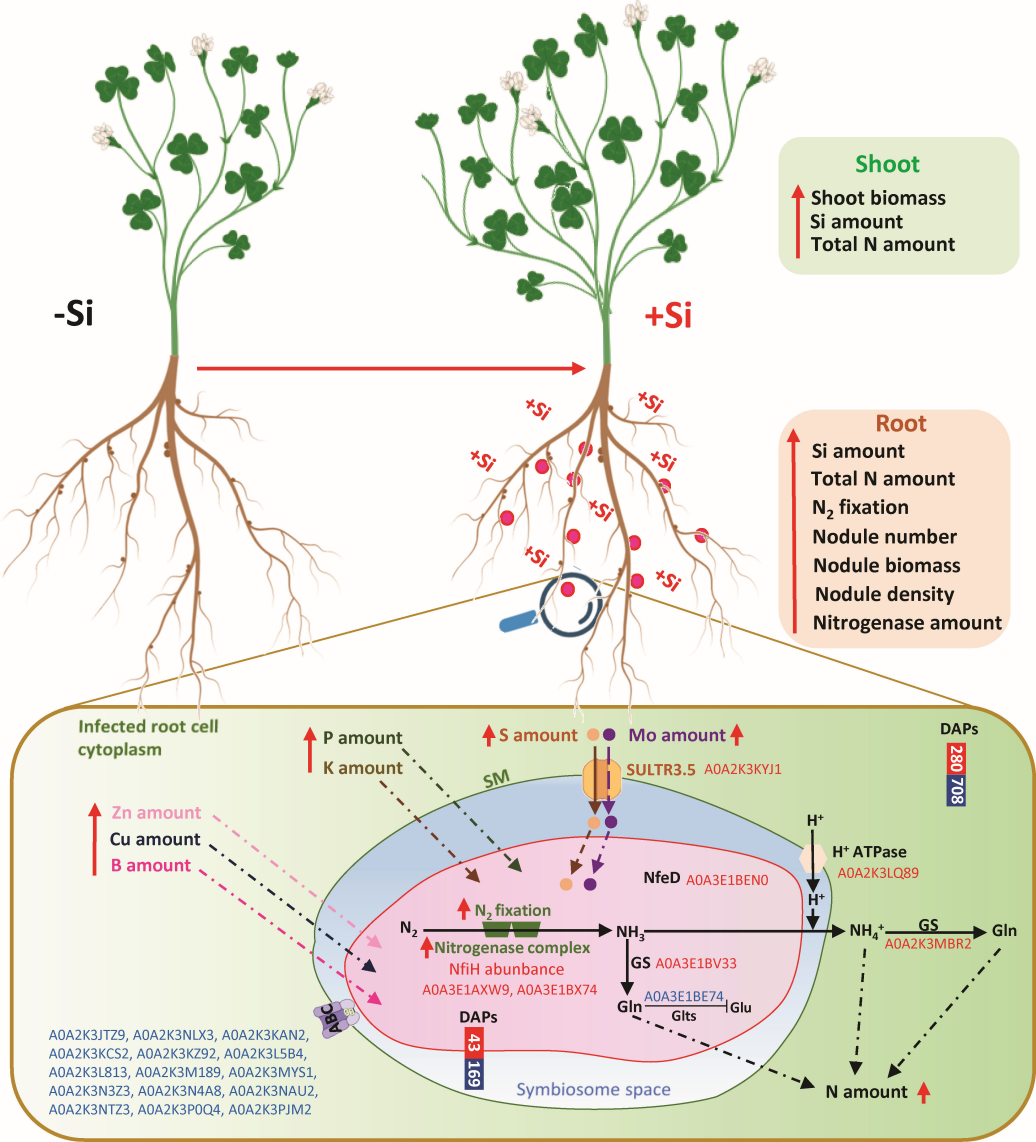
Summary diagram of the main effects of the Si supply on plant growth and nodule parameters in *Trifolium incarnatum* L. Red arrows indicate an increase in the parameters measured in this study. Red and blue Uniprot IDs (**Supplementary Data SD1**) correspond to proteins that are up- and down-accumulated in response to Si supply, respectively. The enlargement corresponds to nodules including infected root cells of *Trifolium incarnatum* L. and the symbiosome including bacteria (*Rhizobium leguminosarum* bv *trifolii*). ABC, ABC transporter cassette; DAPs, differentially accumulated proteins (up- and down-accumulated in red and blue, respectively); GS, glutamine synthase; GltS, glutamate synthase; Gln, glutamate; Glu, glutamine; NifH, iron protein subunit of the bacterial Fe-Mo-co type nitrogenase; NfeD, protein involved in nodulation efficiency (García-Rodríguez and Toro, 2000); SM, symbiosome membrane; SULTR3.5, sulfate transporter homologue to SST1 (Schneider et al., 2019).

The proteomic analysis carried out during this study enabled identification of a total of 8499 proteins in the nodules, of which 6993 (82.3%) belonged to the infected host root cells of *Trifolium incarnatum* L. and 1506 (i.e. 17.7%) belonged to the bacteria (**Figures 4A**, **5A**). This representativeness of both proteomes in nodules is consistent with results reported for nodules of *Medicago truncatula* (Thal et al., 2018). Comparative analysis of the proteomes from nodules of +Si and -Si plants (+Si *versus* -Si; control) revealed that Si supply modulates the proteomes of both infected host root cells and bacteria because 788 (280 up and 708 down) and 212 (43 up and 169 down) DAPs belonged to the infected root cells and bacteria, respectively (**Figures 4A**, **5A**). The GO enrichment analysis with the 788 DAPs from infected root cell revealed a large number of GO terms that were significantly enriched (373, 216 and 122 for BP, MF and CC categories, respectively). Despite this wide dispersion, it is noteworthy that the most enriched GO terms in the BP and MF categories were related to the synthesis of organic nitrogen compounds, amides and peptides (**Figure 4B**). These results are in line with the increase in the amount of N in nodules (**Figures 2A**) but also with previous studies reporting that peptides (such as CLE peptides) and amides (asparagine in particular) are able to enhance the growth and activity of nodules (Sulieman and Tran, 2013). For the CC Go category, one of the most enriched terms was related to “organelle bound” suggesting that some DAPs could be associated with the symbiosome membrane, which is defined as a physical barrier controlled by the plant that regulates ions and organic compounds exchanges between the symbionts (Clarke et al., 2014). Among transporters identified in this membrane, the ABC transporter family is known to participate in the transfer of ions (such as Mo and Mn) and amino acids from the cytoplasm of infected root cells to the symbiosome (Udvardi and Poole, 2013). However, in this study we identified 15 members of the ABC transporter family that are all down-accumulated by Si (**Supplementary Data SD1**, **Supplementary Table SD2**). In the literature, ABC transporters are well known to be upregulated in response to numerous biotic and abiotic stresses (such as salinity, drought and nutrient deficiency) (Li et al., 2021; Kou et al., 2024). In our study, it is possible that the difference in ABC transporter accumulation can be explained by stress attenuation by Si supply, as reported in many studies (Haddad et al., 2018; Ali et al., 2020; Araújo et al., 2022). More interestingly, an increase in the total S amount in nodules was observed in our study and proteome analysis revealed an accumulation of the SULTR3.5 transporter (A0A2K3KYJ1, **Supplementary Data SD1** and **Figure 6**). This SULTR3.5 transporter is a homologue of the SST1 transporter known to be located in the symbiosome membrane that ensures the transport of sulfate from the cytoplasm of the infected root cell to the symbiosome (Schneider et al., 2019). As previously described in *Trifolium repens* (Varin et al., 2010), the increase in the amount of S in the nodule (**Figure 2**) and its possible transport *via* the SULTR3.5 transporter could explain the increase in nitrogenase abundance observed in +Si plant nodules (**Table 1**). In addition, because an earlier study reported that sulfate transporters are also able to transport Mo (Maillard et al., 2016), it cannot be excluded that the SULTR3.5 transporter could be also involved in transporting Mo to the symbiosome. Notably, many studies have reported that Mo and S are both crucial elements for the synthesis of three metal prosthetic groups ([4Fe-4S], P-cluster and FeMo-co) required for synthesis and activity of the nitrogenase complex in bacteria (Schneider et al., 2019; Lilay et al., 2024). This assumption is supported by the up-accumulation of an iron-sulfur cluster carrier protein named NBP35 (A0A3E1BX74, **Supplementary Data SD1**; **Figure 6**), which is involved in the biogenesis of the FeMo-co cofactor (Pardoux et al., 2019). In addition, proteome analysis of the bacteria revealed an accumulation of FixC and NfeD proteins (A0A3E1AXW9 and A0A3E1BEN0, **Supplementary Data SD1**; **Figure 6**), known to provide the reducing power required for nitrogenase functioning (Garcia Costas et al., 2017; Del Cerro et al., 2019) and associated with nodulation efficiency (García-Rodríguez and Toro, 2000), respectively. Taken together, these data are consistent with the enhanced nitrogenase abundance and nitrogen-fixing capacity observed in nodules of +Si plants (**Table 1**, **Figure 1D**), leading to high ammonia production, which could be assimilated via two pathways. Firstly, a portion of the ammonia could be directly assimilated by glutamine synthase (A0A3E1BV33, **Supplementary Data SD1**; **Figure 6**), which is up-accumulated in the cytoplasm of the bacteria. Since glutamate synthase (GltS, A0A3E1BE74) is down-accumulated in the bacteria of nodules in +Si plants, it can be assumed that in this condition, glutamine is either used directly for bacterial protein synthesis or exported to the cytoplasm of the infected root cells. The second and most classical pathway described in the literature (Udvardi and Poole, 2013) consists of exporting ammonia to the symbiosome space, where its protonation to ammonium might be facilitated by the high concentration of H^+^ observed to be accumulated under +Si conditions by the ATPAse H^+^ pump (A0A2K3LQ89, **Supplementary Data SD1**; **Figure 6**) located in the symbiosome membrane. Finally, ammonium exported to the cytoplasm of infected root cells can be assimilated into glutamine by the cytoplasmic glutamine synthase (A0A2K3MBR2, **Supplementary Data SD1**; **Figure 6**), which is also up-accumulated in response to Si. All these results are consistent with the increase in glutamine content observed in nodules of *Medicago truncatula* treated with Si (Putra et al., 2021; 2022), and could explain the significant increase in the amount of N in the nodules of +Si plants, as shown in our study (**Figure 2**).

## 5 Conclusion

This study demonstrates that Si supply increases the nodulation and N_2_-fixation capacity of *Trifolium incarnatum* L. By combining multilevel approaches such as physiological, ionomic and proteomic approaches focused on the nodule, this work provides new insight on the effects of Si and paves the way for a better understanding of the impact of Si on improving nodule function, and more specifically, on the N_2_-fixing capacity of nodules. For the first time, the proteomic approach distinguished between the effects of Si on both symbionts (i.e. infected host root cells and bacteria) and provided a new dataset on the DAPs present in the two nodule partners, which is now available to the scientific community working on Si as well as those seeking to improve the agronomic performance of the Fabaceae.

## Data Availability

The datasets presented in this study can be found in online repositories. The names of the repository/repositories and accession number(s) can be found below: iProX data-set identifier IPX0008420001.
