# Supplementary material for: Ionomic and proteomic changes highlight the effect of silicon supply on the nodules functioning of Trifolium incarnatum L
Source: Front Plant Sci. 2024 Nov 6;15:1462149. doi: 10.3389/fpls.2024.1462149 (PMC11576322; doi:10.3389/fpls.2024.1462149)
Supplement: Supplementary file 2 [file Table1.docx]

Supplementary Material

# Supplementary Tables

**Table SD1:** Macro (S, P, K, N, Ca and Mg) and micro- (Cu, Mo, Fe, Zn, Mn, B and Co) element concentrations in nodules of *Trifolium incarnatum* L. supplied (+Si) or not (-Si) with Si during 25 days. Asterisk indicates that means ± S.E. (n=4) are significantly different between both Si treatment (p< 0.05).

|  |  | **Concentration (µg g^-1^ FW)** | |
| --- | --- | --- | --- |
|  |  | **-Si** | **+Si** |
| **Macroelements** | **S** | 1439.2 ± 61.1 | 1290.4 ± 73.4 |
|  | **P** | 1218.8 ± 82.4 | 1174.1 ± 74.3 |
|  | **K** | 4617.6 ± 178.3 | 4947 ± 159.8 |
|  | **N** | 189.8 ± 8.5 | 324.4 ± 36.5* |
|  | **Ca** | 579.9 ± 36.4 | 474.1 ±41.8* |
|  | **Mg** | 357.5 ± 42.8 | 247.8 ±24.6* |
| **Microelements** | **Cu** | 4.4 ± 0.5 | 5.8 ± 0.6 |
|  | **Mo** | 39.1 ± 3.1 | 38.5 ± 2.4 |
|  | **Fe** | 61.5 ± 0.6 | 59.0 ± 3.2 |
|  | **Zn** | 15.47 ± 3.2 | 22.13 ± 3.3 |
|  | **Mn** | 7.9 ± 0.9 | 7.4 ± 0.7 |
|  | **B** | 2.1 ± 0.2 | 1.9 ± 0.1 |
|  | **Co** | 0.5 ± 0.1 | 0.3 ± 0.1 |

**Table SD2:** List of 1 of 15 ABC transporters down-regulated by Si in root infected cell.

| **UNIPROT** | **Organisme** | **logFC** | **pvalue** | **FDR** | **Expression** | **Protein Name from Uniprot** |
| --- | --- | --- | --- | --- | --- | --- |
| A0A2K3KZ92 | Trifolium | -0.67 | 0.01854464 | 0.738 | Down-regulated | ABC transporter F family member 3-like protein |
| A0A2K3N3Z3 | Trifolium | -0.67 | 0.02208095 | 0.764 | Down-regulated | ABC transporter F family member 3-like protein |
| A0A2K3MYS1 | Trifolium | -0.83 | 0.02487539 | 0.78 | Down-regulated | ABC transporter C family member 4-like protein |
| A0A2K3M189 | Trifolium | -0.95 | 0.04615439 | 0.849 | Down-regulated | ABC-type xenobiotic transporter (EC 7.6.2.2) |
| A0A2K3L5B4 | Trifolium | -1.04 | 0.01129526 | 0.675 | Down-regulated | ABC transporter C family member 8-like protein |
| A0A2K3NAU2 | Trifolium | -1.19 | 0.0292317 | 0.801 | Down-regulated | ABC transporter B family member 19-like protein |
| A0A2K3NTZ3 | Trifolium | -1.20 | 0.00979123 | 0.662 | Down-regulated | ABC transporter G family member 31-like protein |
| A0A2K3P0Q4 | Trifolium | -1.20 | 0.0322849 | 0.812 | Down-regulated | ABC transporter A family member 1-like protein |
| A0A2K3N4A8 | Trifolium | -1.21 | 0.01867853 | 0.739 | Down-regulated | ABC1 family protein |
| A0A2K3JTZ9 | Trifolium | -1.59 | 0.00168072 | 0.43 | Down-regulated | ABC transporter B family member |
| A0A2K3L813 | Trifolium | -1.77 | 0.00183526 | 0.444 | Down-regulated | ABC-type xenobiotic transporter (EC 7.6.2.2) |
| A0A2K3KAN2 | Trifolium | -1.86 | 0.00746742 | 0.634 | Down-regulated | ABC transporter C family member 3-like protein |
| A0A2K3PJM2 | Trifolium | -2.17 | 0.00010561 | 0.121 | Down-regulated | ABC-type xenobiotic transporter (EC 7.6.2.2) |
| A0A2K3NLX3 | Trifolium | -2.23 | 0.00014332 | 0.139 | Down-regulated | ABC transporter B family member |
| A0A2K3KCS2 | Trifolium | -2.39 | 0.00026773 | 0.181 | Down-regulated | ABC transporter B family member |
